# Knowledge, Awareness, and Understanding of Pediatric Triage Among Nursing Officers in India: A Multicenter Study

**DOI:** 10.7759/cureus.46102

**Published:** 2023-09-27

**Authors:** Varun Anand, Chandan K Dey, Arvind Shukla, Murugan TP, Pugazhenthan T, Santosh K Rathia, Sandeep Barman, Anil Kumar Goel, Niraj K Srivastava, Harish Meena

**Affiliations:** 1 Department of Trauma and Emergency, All India Institute of Medical Sciences, Raipur, IND; 2 Department of Community and Family Medicine, All India Institute of Medical Sciences, Raipur, IND; 3 Department of Pediatrics, All India Institute of Medical Sciences, Raipur, IND; 4 Department of Pharmacology and Therapeutics, All India Institute of Medical Sciences, Raipur, IND; 5 Department of Pediatrics, Nagaon Medical College and Hospital, Nagaon, IND; 6 Department of General Surgery, All India Institute of Medical Sciences, Raebareli, IND; 7 Department of Nursing, All India Institute of Medical Sciences, Raipur, IND

**Keywords:** awareness, knowledge, nursing officers, pat, pediatric assessment triangle, pediatric triaging, ed, emergency department, triage

## Abstract

Introduction: Triage is crucial in patient screening within emergency departments (EDs) worldwide. It is one of the essential and standard medical practices in many developed countries. However, in India, there is a need for improvement in triage utilization, as it is predominantly performed by resident doctors or medical officers, leading to an uneven distribution of clinical skills among healthcare providers (HCPs). A comprehensive analysis incorporating literature review and data collection revealed that while mandatory screening is conducted in most Indian EDs, the formal implementation of standardized triage protocols remains limited. Like in developed countries, registered nurses or nursing officers (NOs) can be effectively trained and directed to play the role of dedicated triage personnel in EDs of most of the healthcare facilities in India.

Method and materials: This study aimed to examine the current state of triage utilization and its impact on the distribution of responsibilities among HCPs in Indian EDs. Through this online survey, the investigators assessed the knowledge and practical understanding of clinical triaging among NOs, working at various hospitals nationwide.

Results: The participants included 5,029 NOs working in various parts of India, predominantly nursing graduates (82.52%), the majority being employed in government healthcare settings (84.01%) and most having over five years of cumulative working experience in the ED (70.77%). Nurses showed inadequate knowledge and awareness about the Pediatric Assessment Triangle (PAT) used for quick initial evaluation (62.18% among all participants). Concerning the complete triage process applicable, especially in pediatric ED settings, they had even less satisfactory knowledge and understanding, e.g., identifying primary (28.27%) and secondary (22.69%) survey components via focused history and examination, properly using temperature assessment (23.32%) and instant blood glucose level assessment (22.95%) in triage, and knowing various types of internationally accepted triage systems for ED-based health facilities such as the Emergency Severity Index (ESI), Canadian Triage and Acuity Scale (CTAS), and Australasian Triage Scale (ATS) (15.87%).

ANOVA and post hoc analysis revealed that the intergroup performance of the study participants with maximum correct responses to the knowledge-determining specified subset of the questionnaire depicts the significantly higher role of graduate nursing degree over diploma such as General Nursing and Midwifery (GNM)/Auxiliary Nursing and Midwifery (ANM) qualification, working in government hospital versus private setup, and ED working experience of >5 years over that of <5 years.

Conclusions: Of the participants in the study, 50% were not evaluated for cognitive or psychomotor domains during their assessment examinations. The research illuminated a significant disparity in knowledge and proficiency levels among Indian nurses concerning pediatric triage, especially with the ability to effectively apply the PAT for initial patient evaluations, discern components of primary and secondary surveys, and comprehend various triage systems.

This study underscores the importance of comprehensive reform in the Indian healthcare system and teaching curriculum by emphasizing clinical triage training and interprofessional collaboration, and establishing guidelines and regulations to ensure consistent and standardized triage practices across all EDs.

## Introduction

Triage is an algorithm-based systematic approach of quick evaluation done on all emergency patients that guides the clinical assessor to prioritize them based on the severity and acuity of illness to select the required interventions and treatment [1]. Thus, in situations such as mass causality and disasters, triaging becomes the utmost required tool for sorting out the most critical emergencies and appropriately allocating available resources to fetch the best possible preeminent outcome. Pediatric triaging is a systematic process incorporating a swift initial assessment utilizing the Pediatric Assessment Triangle (PAT), which relies on quick visual and auditory perception. Ideally completed within 30-50 seconds, this initial assessment identifies life-threatening conditions in any sick patient [2].

Since pediatric emergency medicine (PEM) is a branch that has yet to develop in all health facilities in India, pediatric emergency cases are either assessed at pediatric general wards or adult emergency departments (EDs). All healthcare providers (HCPs) must be acquainted with pediatric triage skills, as it helps optimize hospital resources, especially during disease epidemics and mass disaster incidents [3]. Triaging helps decrease the left-without-being-seen (LWBS) rate and length of stay (LOS) in the ED.

In India, nursing officers (NOs) are not well-trained and skilled in pediatric triage, whereas in developed countries such as the USA, Australia, the UK, and Canada, triage is carried out by specialized triaging officers who possess qualifications equivalent to a registered nurse or nursing officer, and they are specifically trained in triaging skills. Integrating real-patient triage training into the undergraduate nursing practical curriculum can generate a substantial workforce capable of performing triage efficiently, particularly in situations involving pandemics and mass casualties [4].

According to a cross-sectional study, nurses and nursing students should undergo comprehensive training and reinforcement to acquire proficient triaging skills [5]. A systematic review revealed a notable paucity of research on evaluating pediatric triage tools in low- and middle-income countries (LMICs) [6,7]. The present study involved the assessment of pediatric triage knowledge and skills among participating nursing officers nationwide. The objective of the current study was to assess the knowledge and proficiency of nursing officers in accurately determining the acuity and treatment prioritization in children presenting to the emergency room by utilizing the concept and protocols of pediatric triage.

## Materials and methods

Prior to the commencement of the study, necessary clearance was obtained from the Institutional Research Cell (IRC) and the Institutional Ethics Committee (IEC) (approval number: 1268/IEC-AIIMSRPR/2020), ensuring compliance with ethical guidelines and research protocols. The study’s inclusion criteria were registered nurses with educational qualifications, including diplomas, graduation, and postgraduate degrees, working in India in all healthcare setups, including the private sector. Incomplete survey forms were excluded from the study. The study was conducted at the All India Institute of Medical Sciences (AIIMS), Raipur, where predetermined criteria (Table 1) were established for developing a questionnaire to evaluate the knowledge, awareness, and understanding of nursing officers regarding pediatric triaging.

**Table 1:** Criteria for finalizing the questionnaire MCQ: multiple-choice question, PAT: Pediatric Assessment Triangle

Serial number	Criteria of questionnaire	Checklist
1	The questionnaire is well-readable and has sufficient space between the questions.	
2	The font size of the questionnaire is easily readable.	
3	The questionnaire has sufficient space to answer.	
4	All questions have multiple choice-based answers (MCQ) to select.	
5	All questions in the questionnaire must be answerable with a single option.	
6	All questions are well-framed and have clear meaning to understand.	
7	The grammar of all questions is correct.	
8	There is no spelling mistake in the questionnaire.	
9	The questionnaire includes an assessment of the PAT.	
10	The questionnaire includes an assessment of the airway.	
11	The questionnaire includes an assessment of breathing.	
12	The questionnaire includes an assessment of circulation.	
13	The questionnaire includes an assessment of disability (central nervous system abnormality).	
14	The questionnaire includes an assessment of the requirement of blood dextrose level.	
15	The questionnaire includes an assessment of exposure.	
16	The questionnaire includes an assessment of temperature.	
17	The questionnaire includes an assessment of the interventions that may be required in between the steps of the primary survey.	
18	The questionnaire includes an assessment of the secondary survey.	
19	The questionnaire includes an assessment of proper triage labeling.	
20	The questionnaire includes an assessment of trauma or injuries.	
21	All questions are valid for the study and assess the knowledge, awareness, and understanding of participants in pediatric triaging skills.	

A meticulously crafted and comprehensive questionnaire was subsequently formulated by clinical experts specializing in pediatric emergency medicine (PEM) at AIIMS, Raipur, with a focus on assessing nursing officers’ practical understanding of PAT assessment, primary survey, required adjuncts and interventions, secondary survey, and proper labeling of triage level.

To ensure the validity of the questionnaire, a rigorous validation process was undertaken, involving local proofreading and two separate clinical experts with expertise in PEM or pediatric triaging. Utilizing the Delphi method through electronic communication, the experts were engaged in a blinded exchange of comments and recommendations on improvising the questionnaire, reiterating this process twice to arrive at a consensus for final validation. The questionnaire was deemed finalized upon mutual agreement between the independent experts, guaranteeing its robustness and reliability.

For effective coordination and guidance to ensure the vast reach of the study’s invitation links and completion of the questionnaire by maximal participants, the responsibility was assigned to linking coordinators from each geographical zone (north, south, east, west, and central) across the country, thereby ensuring comprehensive coverage and robust representation of nursing officers.

The questionnaire was uniformly distributed to target participants, including nursing officers and staff nurses, through electronic media utilizing a web link. Upon clicking the web link, a dedicated web page was accessed, taking the participants to a consent form that they had to read and acknowledge by clicking on it.

Statistical analysis

The data from the online survey was translated into an Excel spreadsheet (Microsoft Corporation, Redmond, WA), and participants giving incomplete answers were excluded. For statistical analysis, Statistical Package for the Social Sciences (SPSS) version 20 (IBM SPSS Statistics, Armonk, NY) was used. For frequency and percentage data, Chi-square tests were used. Data was collected through 25 questions in the “online questionnaire form.” Marks obtained by the participants based on the subset of 15 questions targeted to assess knowledge were utilized as a percentage score for subgroup analysis using an analysis of variance (ANOVA) test. Post hoc analysis was conducted to determine any significant intergroup differences (p-value of <0.05).

## Results

The initial enrollment in this questionnaire-based study comprised a total of 5,089 participants. However, after excluding 60 responses with incomplete data, the final analysis included 5,029 responders. The participants were NOs and comprehensively represented diverse geographical regions, including prestigious institutes of national importance (INIs), prominent government medical colleges, and many private hospitals across India.

The knowledge and triage proficiency was evaluated using a predetermined set of 15 out of 25 questions encompassed in Google Forms. Subgroup analysis was done on the participants’ performance based on their score percentage concerning their workplace setting (government or private hospital), educational qualifications (General Nursing and Midwifery (GNM)/Auxiliary Nursing and Midwifery (ANM) diploma, bachelor in nursing, and postgraduate in nursing), and cumulative working experience levels at the ED (zero, <1 year, 1-5 years, and >5 years), as detailed in Tables 2-4.

**Table 2:** Knowledge and understanding of the triage system based on institutional preference PAT: Pediatric Assessment Triangle, ED: emergency department, ICD: intercostal drainage, ECG: electrocardiogram, SVT: supraventricular tachycardia

Important parameters in the questionnaire	Government sector (n=4,225) (number (%))	Private sector (n=804) (number (%))	p-value
Worked in dedicated pediatric emergency	2,871 (68)	471 (58.6)	0.000
Worked as a triage nurse	2,270 (53.7)	470 (58.5)	0.007
Knew the process of triaging	2,697 (63.8)	361 (44.9)	0.000
Got the questions related to triage during the theory examination	2,314 (54.8)	458 (57)	0.134
Assessed for practical aspects of triage during training	2,634 (62.3)	404 (50.2)	0.000
Knowledge of airway as the first assessment during triage	1,227 (29)	195 (24.3)	0.000
Done PAT assessment in clinical settings	2,669 (63.2)	458 (57)	0.001
Understanding of PAT assessment	1,037 (24.5)	211 (26.2)	0.306
Correct order of initial assessment at the ED	2,091 (49.6)	281 (38.5)	0.000
Components of the secondary survey	985 (23.3)	156 (19.4)	0.000
Measurement of temperature during the primary survey	936 (22.2)	237 (29.5)	0.000
Checking of glycemic level in primary survey	933 (22.1)	221 (27.5)	0.000
Timing of intubation during triage and assessment	1,190 (28.2)	269 (33.5)	0.000
Triage level of a child in active seizure	1,249 (29.6)	113 (14.1)	0.000
ICD insertion for pneumothorax as an emergency procedure	1,615 (38.2)	237 (29.5)	0.000
Timing of oxygen therapy during active seizures	758 (17.9)	128 (15.9)	0.172
Importance of ECG in a sick child	1,784 (42.2)	408 (50.7)	0.000
Management of SVT in the ED	2,317 (54.8)	277 (34.5)	0.000

**Table 3:** Knowledge and understanding of the triage system based on educational qualification PAT: Pediatric Assessment Triangle, ED: emergency department, ICD: intercostal drainage, ECG: electrocardiogram, SVT: supraventricular tachycardia

Important parameters in the questionnaire	Graduate (n=4,150) (number (%))	ANM/GNM (n=462) (number (%))	Postgraduate (n=417) (number (%))	p-value
Worked in dedicated pediatric emergency	2,821 (68)	264 (57)	257 (61.6)	0.000
Worked as a triage nurse	2,232 (53.8)	276 (59.7)	232 (55.6)	0.045
Knew the process of triaging	2,664 (64.2)	230 (49.8)	164 (39.3)	0.000
Got the questions related to triage during the theory examination	2,284 (55)	226 (56.7)	262 (54.2)	0.731
Assessed for practical aspects of triage during training	2,581 (62.2)	233 (50.4)	224 (53.7)	0.000
Knowledge of airway as the first assessment during triage	1,197 (28.8)	138 (29.9)	87 (20.9)	0.000
Done PAT assessment in clinical settings	2,623 (63.2)	253 (54.38)	251 (60.2)	0.001
Understanding of PAT assessment	1,039 (25)	142 (30.7)	127 (30.5)	0.002
Correct order of initial assessment at the ED	2,062 (49.7)	191 (41.3)	125 (30.6)	0.000
Components of the secondary survey	975 (23.5)	94 (20.3)	72 (17.3)	0.000
Measurement of temperature during the primary survey	922 (22.2)	129 (27.9)	122 (29.3)	0.000
Checking of glycemic level in primary survey	919 (22.1)	114 (24.7)	121 (29)	0.000
Timing of intubation during triage and assessment	1,174 (28.3)	140 (30.3)	145 (34.8)	0.000
Triage level of a child in active seizure	1,208 (29)	82 (17.7)	75 (18)	0.000
ICD tube insertion for pneumothorax as an emergency procedure	1,587 (38.2)	156 (33.2)	109 (26.1)	0.000
Timing of oxygen therapy during active seizures	710 (17.1)	89 (19.3)	87 (20.9)	0.000
Importance of ECG in a sick child	1,732 (41.7)	238 (51.5)	222 (53.2)	0.000
Management of SVT in the ED	2,275 (54.8)	173 (37.4)	146 (35)	0.000

**Table 4:** Knowledge and understanding of the triage system based on working experience PAT: Pediatric Assessment Triangle, ED: emergency department, ICD: intercostal drainage, ECG: electrocardiogram, SVT: supraventricular tachycardia

Important parameters in the questionnaire	<1 year (n=508) (number (%))	1-5 years (n=3,559) (number (%))	>5 years (n=492) (number (%))	p-value
Worked in dedicated pediatric emergency	304 (59.8)	2,492 (70)	275 (55.9)	0.000
Worked as a triage nurse	316 (62.2)	1,853 (52.1)	285 (57.9)	0.000
Knew the process of triaging	244 (48)	2,369 (66.6)	219 (44.5)	0.000
Got the questions related to triage during the theory examination	289 (56.9)	1,912 (53.7)	289 (58.7)	0.014
Assessed for practical aspects of triage during training	263 (51.8)	2,254 (63.3)	265 (53.9)	0.000
Knowledge of airway as the first assessment during triage	151 (29.7)	1,003 (28.2)	113 (23)	0.000
Done PAT assessment in clinical settings	288 (56.7)	2,300 (64.6)	263 (53.5)	0.000
Understanding of PAT assessment	143 (28.1)	887 (24.9)	146 (29.7)	0.000
Correct order of initial assessment at the ED	177 (34.8)	1,881 (52.9)	146 (29.7)	0.000
Components of the secondary survey	86 (16.9)	863 (24.2)	91 (18.5)	0.000
Measurement of temperature during the primary survey	165 (32.5)	744 (20.9)	144 (29.3)	0.000
Checking of glycemic level in primary survey	137 (27)	757 (21.3)	149 (30.3)	0.000
Timing of intubation during triage and assessment	166 (32.7)	977 (27.5)	166 (33.7)	0.000
Triage level of a child in active seizure	108 (21.3)	1,107 (31.1)	59 (12)	0.000
ICD tube insertion for pneumothorax as an emergency procedure	169 (33.3)	1,384 (38.7)	147 (29.9)	0.000
Timing of oxygen therapy during active seizures	104 (20.5)	610 (17.1)	89 (18.1)	0.000
Importance of ECG in a sick child	270 (53.1)	1,423 (40)	264 (53.7)	0.000
Management of SVT in the ED	192 (37.8)	2,048 (57.5)	182 (37)	0.000

Workplace (government versus private)

Among the total participants, a substantial majority (84.01%, n=4,225) were employed in government-based health sectors, with a considerable proportion (67.95%, n=2,871) having focused experience working in pediatric emergency facilities. In contrast, a smaller percentage of participants (15.99%, n=804) were associated with private hospitals; of them, 58.58% (n=471) had exposure to pediatric emergency services.

Educational qualification

**Figure 1:**
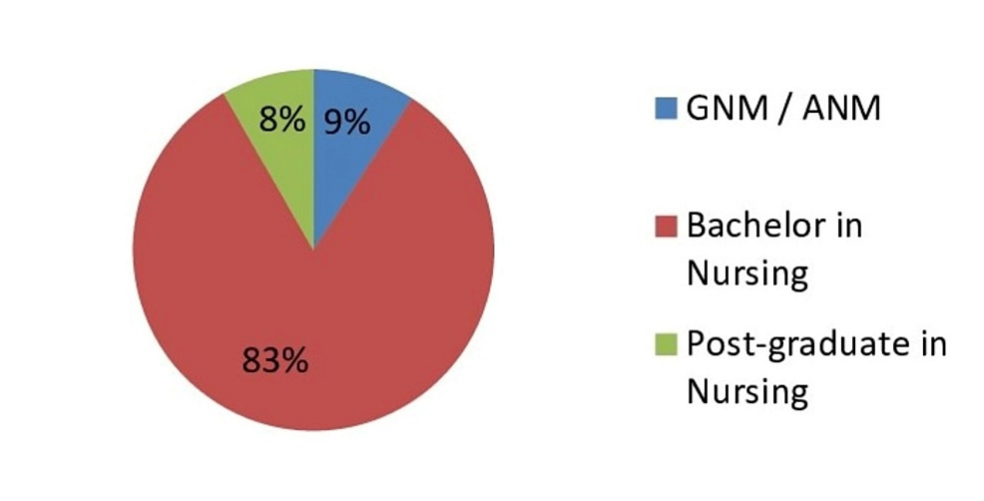
Educational qualification of the study participants GNM: General Nursing and Midwifery, ANM: Auxiliary Nursing and Midwifery

Of all the participants, a majority had a professional qualification with a bachelor’s degree in nursing (82.52% of total participants, n=4,150), and the rest had a nursing diploma, with a few having a postgraduate degree in nursing (Figure 1).

Among the 3,342 participants having experience in pediatric emergency, the majority (85%) held a Bachelor of Science (BSc) degree in their nursing qualification. Additionally, an analogous number of participants had either a diploma in General Nursing and Midwifery (GNM)/Auxiliary Nursing and Midwifery (ANM) (7.89%) or a postgraduate nursing degree (7.69%). The differences in qualifications between graduate, diplomas, and postgraduate degrees were statistically significant.

Working experience

**Figure 2:**
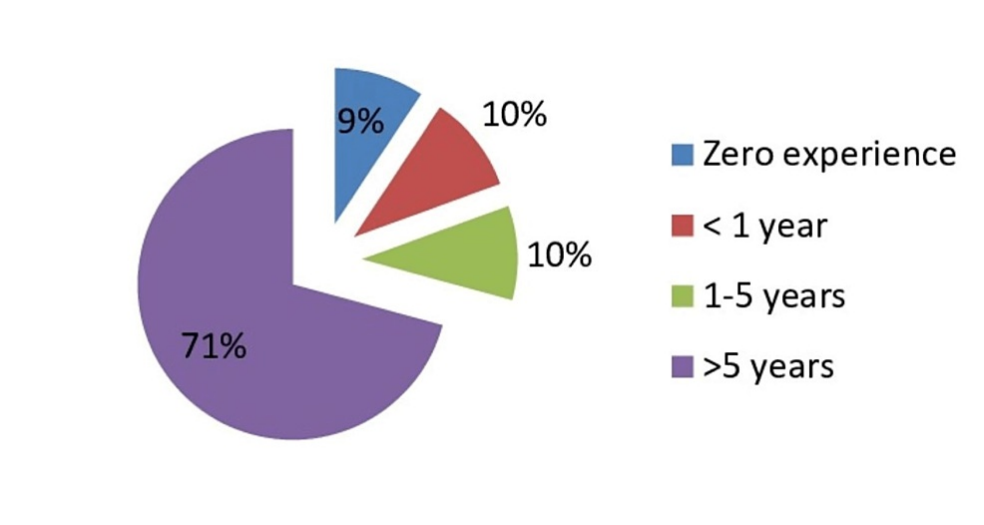
Experience of working at any emergency department or casualty

The majority of the study participants had experience working at any ED or casualty for a cumulative duration of more than five years (70.77%, n=3,559), and out of them, 2,492 (70.02%) also had experience working in any of the pediatric emergency facility (Figure 2).

A notable proportion of all participants (66.45%, n=3,342) had clinical experience managing pediatric emergencies. Out of them, 75% had more than five years of experience working in any emergency or casualty.

Exposure to ED working and hospital-based triaging practice

Our findings revealed that approximately half of the participating NOs (54.48%, n=2,740) had practical exposure to an ED or a hospital setting where the triaging process was implemented in clinical practice. Moreover, a slightly higher proportion (60.80%) acknowledged their familiarity with the triage process and recognized its importance. Among those who reported having worked in an actual triage-based health facility, the majority were employed in government sectors (88.19%, n=2,697), demonstrating a higher qualification level (87% held a bachelor’s degree), and possessed substantial clinical experience (78% had worked in an emergency setting for more than five years).

Triaging concepts in teaching curriculum

A questionnaire component was designed to assess participants’ exposure to triage knowledge and practice during their nursing degree or diploma courses, including their assessment in final examinations. Many study participants acknowledged that they had never been assessed for triage-related cognitive and psychomotor aspects (44.88% and 39.59%, respectively) during their training period. Delving into the nursing qualifications of participants who had never been assessed on their knowledge and cognitive understanding about clinical triage at their exit examination, 45% were diploma in nursing (GNM/ANM), 43% were BSc in nursing graduates, and 45.80% possessed postgraduate qualifications.

Awareness of different triage systems

Participants were queried about their familiarity with various triage systems through different sources. The findings revealed that 36.25% of the participants reported being aware of the Emergency Severity Index (ESI) only, 29.45% were acquainted with the Canadian Triage and Acuity Scale (CTAS), and 18.43% were aware of the Australasian Triage Scale (ATS) only. While merely 15.87% of the study participants could correctly identify all three internationally recognized triage systems as applicable to hospital-based or ED settings. The Emergency Severity Index (ESI) received the highest recognition and awareness (52.12% among all participant nurses) compared to other identified triage systems. A majority of these nurses had a BSc in nursing qualification (53.25% among all graduates, n=2,210), worked in government healthcare settings (53.96% of all those working at government setup), and had >5 years of experience working in an emergency setting (53.81% among participants with >5 years experience).

PAT and primary survey

An equal percentage of participants with different educational levels, including diploma holders (74.68%), graduates (71.06%), and postgraduates (73.62%), showed a need for more awareness regarding the PAT and its expansion. The analysis of PAT-related responses indicated that 62.18% of the participants acknowledged utilizing the initial assessment triangle (PAT) while triaging pediatric patients. However, it is noteworthy that only 28.39% of the participants could accurately describe the expansion of PAT as the “Pediatric Assessment Triangle.” Approximately 28.27% (n=1,422) of the participants demonstrated accurate comprehension when asked about the ABCDE elements of the primary survey applied in screening sick patients in an ED.

Airway and intubation interventions

A total of 1,230 (24.46%) participants responded correctly regarding the time-sensitive intervention of maintaining the airway and deciding on the need for intubation during the ABCDE of primary assessment only. This particular knowledge and awareness were better among the nurse officers working in private hospitals (28.11% of total participants working at private setups) than those working in government hospitals (23.76% of total participants working at government setups), and this difference was statistically significant. Notably, it was higher among participants with a nursing diploma (29.22% of total participating diploma nurses) than candidates with a nursing degree qualification.

Active seizure episodes

Only 27.08% of the participants accurately recognized the correct level of triage for a child with active seizures brought to the ED (i.e., triage level 1 and red category). A statistically significant difference was observed in the proportion of participants providing correct answers between government healthcare settings (29.56%) and private healthcare settings (14.05%). Most correct responses (31.10%) came from the highest work experience group.

Oxygen therapy

The analysis of questions regarding oxygen supplementation as a part of the initial lifesaving interventions (LSIs) after the PAT evaluation of a child with active seizures revealed that only a small number of participants (17.62%) answered correctly. A higher proportion of correct responders were among those working at government hospitals (17.94%) and with postgraduate degrees in nursing (20.86%).

Pneumothorax

The participants underwent assessment through a clinical case scenario focused on pneumothorax, wherein the objective was to determine their decision regarding the insertion of intercostal chest tube drainage as a lifesaving intervention (LSI) or emergency procedure. Approximately one-third (36.83%) of the participants expressed a preference for conducting the procedure as an emergency LSI rather than waiting to transfer the patient to the pediatric intensive care unit (PICU), operating theater (OT), or ward. The majority of participants who selected this as an emergency procedure belonged to the government working group (38.22% of the total government working participants), held nursing degrees (38.24% of the total graduate participants), and had over five years of experience in the emergency department (38.89% of the total most experienced participants).

ECG and treatment of SVT

The need for ECG in a cardiac emergency such as supraventricular tachycardia (SVT) during the circulatory assessment and identifying one of the most accurate interventions, i.e., administration of adenosine injection, was assessed. Among government hospital employees, BSc-qualified nursing officers, and those with five years of ED experience, correct response rates were 54.84%, 54.82%, and 57.54%, respectively. More details of assessed parameters are available in Tables 2-4.

Assessment of glucose level

We had asked about the importance of rapid dipstick testing for glucose levels during triage of any sick patient, and only 22.95% of the participants could correctly identify it as an integral part of disability/dextrose assessment in the primary evaluation.

Temperature assessment

Merely 23.32% of the study participants correctly understood the significance of temperature assessment, particularly in pediatric cases. Even among the most experienced group (with over five years of experience in an ED setting), only 20.90% responded accurately to this question. Similarly, only 29.26% of the participants with the highest postgraduate degree in nursing could identify the correct response.

Secondary survey

Only 1,141 NOs (22.69%) knew the components of the secondary survey, viz., focused history and focused examination. Out of them, most were primarily working in government sectors (86.33% of these 1,141 NOs) and were BSc in nursing graduates by qualification (85.45%), and a majority (75.64%) of them had ED experience of >5 years.

Reassessment/re-triaging

Almost 48% of the participants expressed that reassessment is necessary after each step of triage evaluations and interventions in any emergency setting to watch for response and its adequacy to correct the deranged physiology. The rate of correct response was higher among the participants employed at government hospitals (51.86%), holding a BSc degree in nursing (52.41%), and having more than five years of ED experience (56.08%).

Overall performance in the assessment of knowledge

The study questionnaire included a total of 25 questions, out of which 15 questions (question numbers 7, 9, 11, 12, 13, 14, 15, 16, 18, 19, 20, 22, 23, 24, and 25) were predetermined to assess the knowledge of the participants. Table 5 presents key questionnaire parameters for assessing knowledge of pediatric triage.

**Table 5:** Frequency distribution of responses to the questions PAT: Pediatric Assessment Triangle, ED: emergency department, ICD: intercostal drainage, ECG: electrocardiogram, SVT: supraventricular tachycardia

Assessment of important parameters in the questionnaire in the form of “yes”	Number (%)
Worked in dedicated pediatric emergency	3,342 (66.5)
Worked as a triage nurse	2,740 (54.5)
Knew the process of triaging	3,058 (60.8)
Got the questions related to triage in theory examination	2,772 (55.1)
Got assessed for practical aspects of triage during training	3,038 (60.4)
Done PAT assessment in clinical settings	3,127 (62.2)
Assessment of important parameters in the questionnaire in the form of “correct response”	Number (%)
Knowledge of airway as the first assessment during triage	1,422 (28.3)
Understanding of PAT assessment	1,248 (24.8)
Correct order of initial assessment at the ED	2,372 (47.2)
Components of the secondary survey	1,141(22.7)
Measurement of temperature during the primary survey	1,173 (23.3)
Checking of glycemic level in primary survey	1,154 (22.9)
Timing of intubation during triage and assessment at the ED	1,459 (29)
Triage level of a child in active seizure	1,362 (27.1)
ICD tube insertion for pneumothorax as an emergency procedure	1,852 (36.8)
Timing of oxygen therapy during active seizures	886 (17.6)
Importance of ECG in a sick child	2,192 (43.6)
Management of SVT in the ED	2,594 (51.6)

Upon comparing the performance of government and private sector employees, on evaluation of knowledge, the mean percentage score ± standard deviation (SD) for government employees was 30.7±12.9. In contrast, private sector employees had a lower mean score of 29.4%, with a standard deviation of 12.6%, which is statistically significant (Table 6).

**Table 6:** Assessment of knowledge score according to the workplace of the respondent N: number of participants, SD: standard deviation, SE: standard error

Workplaces	Number	Mean (% score)	SD	SE	p-value
Government sector	4,225	30.71	12.9	0.20	0.008
Private sector	804	29.42	12.6	0.44	

In the assessment of participants’ performance on the specified questionnaire for triage knowledge and understanding, we found that participants with a BSc/post-BSc qualification achieved a higher average score of 31%, demonstrating better performance in the triage knowledge assessment compared to both the GNM/ANM group and the postgraduate (nursing) group, which had average scores of 28.76% and 27.37%, respectively (Table 7).

**Table 7:** Scores of respondents on knowledge-based questions in relation to their educational qualification SD: standard deviation, GNM: General Nursing and Midwifery, ANM: Auxiliary Nursing and Midwifery, BSc: Bachelor of Science

Qualification	Mean (% marks obtained)	SD	p-value
GNM/ANM	28.8	14.2	<0.001
BSc	31	12.6	
Postgraduate	27.4	12.9	
Post hoc analysis			
GNM/ANM	28.8	14.2	0.001
BSc	31	12.6	
GNM/ANM	28.8	14.2	0.242
Postgraduate	27.4	12.9	
BSc	31	12.6	0.001
Postgraduate	27.4	12.9	

Upon conducting an in-depth analysis using ANOVA, we discovered significant differences in the participants’ performance based on their qualifications.

Post ANOVA, a post hoc analysis was also conducted to determine the specific intergroup differences concerning nursing qualification. The results revealed statistically significant distinctions between group 1 (GNM/ANM) and group 2 (BSc), with a p-value of <0.001. Similarly, we observed a significant difference between group 1 and group 3 (postgraduation), with a p-value of 0.001. These findings indicate that the variations in performance among the different qualification groups are not due to chance, but rather, they are statistically meaningful (Table 3).

The participants’ performance was analyzed based on their experience level, dividing them into four distinct groups, as presented in Table 4. It was observed that group 1, consisting of participants with no prior experience, achieved a mean score of 29.09%. Meanwhile, the mean scores for the other groups were as follows: group 2 (<1 year of experience) scored 29.38%, group 3 (1-5 years of experience) scored 29.66%, and group 4 (>5 years of experience) scored 31.29%. ANOVA also revealed a significant difference among the four groups of ED work experience (p<0.001). Subsequently, a post hoc analysis was performed for multiple comparisons to delve into the specific intergroup differences, as shown in Table 8, which revealed that participants in group 4 (those with >5 years of experience) performed significantly better than the other three groups.

**Table 8:** Scores of respondents on knowledge-based questions in relation to their work experience in an ED SD: standard deviation, ED: emergency department

Experience	Mean	SD	p-value
Never	29.1	15.1	<0.001
<1 year	29.4	15.9	
1-5 years	27.4	13.5	
>5 years	31.3	11.8	
Post hoc analysis			
Never	29.1	15.1	0.985
<1 year	29.4	15.9	
Never	29.1	15.1	0.162
1-5 years	27.4	13.5	
Never	29.1	15.1	0.003
>5 years	31.3	11.8	
<1 year	29.4	15.9	0.064
1-5 years	27.4	13.5	
<1 year	29.4	15.9	0.009
>5 years	31.3	11.8	
1-5 years	27.4	13.5	<0.001
>5 years	31.3	11.8	

## Discussion

Triage expedites critical patient identification and prioritization in the ED by distinguishing severe cases from nonurgent ones, thereby streamlining patient care [8]. Implementing triage protocols within EDs has demonstrated a substantial decrease in emergency care waiting times and overall patient contentment [9]. Factors such as exactitude, accuracy, and aptness of a triage officer in a triage unit are critical in influencing the ultimate ED outcomes [10]. In developed nations such as the USA, the role of a triage officer is entrusted to individuals possessing qualifications equivalent to a nurse practitioner, backed by adequate clinical experience and specialized training in triage. In contrast, deploying a dedicated triage officer at all EDs in India remains sporadic and insufficient; instead, the responsibility is often delegated to resident doctors in medical colleges and casualty medical officers who are often overburdened with ongoing patient care. Consequently, this disparity results in an inequitable allocation of duties to the health personnel, disproportionately engaging healthcare providers with more specialized expertise with basic-level services such as triaging. Despite notable progressive advancements in various health indicators compared to their pre-independence levels, the doctors-to-patient ratio is still worse than the nurses-to-patient ratio in India [11,12]. In the current study, up to 10% of nursing officers had yet to be exposed to a working environment in an ED setting.

A triage NO should be trained to perform an accurate triage by primary teaching, training, simulation, and workshops, and their knowledge should be monitored periodically, including skill updates by frequent mock drills. In many studies, it has been seen that hours of training of triage officers and continuous dedicated exposure in the ED affect triage accuracy [8-10]. The present study observed a similar trend, wherein individuals with more significant experience in the ED exhibited superior performance in knowledge assessment.

A postal survey-based study conducted in Australasia to evaluate the requisite experience and training necessary for nurses assigned to triage roles involving all EDs of the country found substantial diverse training methods for triage nurses, including charge nurses and unit nurse managers. It was recommended to establish accessible training and minimum standards for designating nurses in triage roles [13].

Ensuring comparable training for nursing officers in private healthcare settings as in government setups is essential; our study also revealed that experienced nursing officers in government hospitals had notably greater exposure to pediatric emergencies than their counterparts from private facilities.

A prior study on senior medical students using a questionnaire that included different case scenarios revealed worrisome inadequacy in triage knowledge and decision-making. Consequently, a strong recommendation was made to revise educational courses to enhance comprehension of triage practices [14]. In the current study, the comprehension of the triage process and its inferential application for clinical practice by nursing officers needed to be improved, particularly in clinical case-based scenarios. The exploration of triage knowledge has been the subject of previous investigations conducted among diverse healthcare professionals [15-18].

In a cross-sectional analytical questionnaire-based study involving nursing and emergency medical service students, it was deduced that their knowledge and understanding of triage in the ED were deficient, and it was likely attributable to inadequate triage education courses, including practical training [19]. The current study demonstrated that nearly 40%-50% of participants did not encounter any questions on triaging in their examinations, which was alarming. Restructuring of nursing curriculum might help improve this aspect. Collective paper-based case scenarios and high-fidelity simulation approaches in triage teaching are better than individual types of teaching methodology [7]. As emergency nurses play a vital role in prioritizing triage, it is imperative for nursing education programs to include dedicated courses on triage to ensure mastery of this crucial aspect of their practice.

Although more than 60% of the participating nurses in the current study acknowledged familiarity with the triage system, nearly half of the participants had never worked as a triage officer in any clinical setting. The CTAS, ESI, and Manchester Triage System (MTS) are the most accepted triage systems worldwide, but the current study revealed that nearly 70% of the participants were unaware that all these systems were used for triage. The ESI system was the most known triage system, and that too was known to only about half of the study participants, of which the majority were graduates and had >5 years of experience. Multiple studies conducted in different countries utilizing various triage systems, such as the ESI, Emergency Triage Assessment and Treatment (ETAT), and CTAS, consistently concluded that nurses exhibited inadequate knowledge in accurately determining triage levels. Consequently, a common recommendation emerged across these studies, emphasizing the need to strengthen triage training and education [20-22].

Findings from another study in Hong Kong shed light on the importance of developing formal triage training programs and implementing standardized ED protocols to assist prompt decisions on triage level allocation, especially for trauma and emergency cases. The study also underscored the significance of providing positive reinforcement and robust support to triage nursing officers to enhance their competency in making effective and timely triage decisions [23].

The current study has a similar pattern of lack of awareness in triage among nurses and therefore recommends that nursing officers undergo targeted training during their educational programs and practical assignments at EDs. This approach may provide them invaluable opportunities to gain first-hand experience in patient triage scenarios, potentially enhancing their confidence and aptitude in effectively managing mass casualties and responding to diverse disaster situations. The current study found that more than two-thirds of the nurses had problems identifying the correct triage level, performing life-saving procedures at the ED, reassessing the emergency patients, and implementing interventions after the initial impression and during triage. Less than 40% of the participants knew that triage is done by a person whose qualification is equivalent to nursing with experience in triage training. It means that understanding of the practical need of doing triage could have been better among participating nurses.

A Swedish study conducted across 48 EDs involving 423 registered nurses assessed the accuracy of identifying clinical triage scenarios using the Canadian Triage and Acuity Scale (CTAS). The findings revealed that approximately 58% of registered nurses successfully assigned accurate acuity ratings, comparable to the current study, where the identification of the correct triage level in the questionnaire of the current study was done by 27.08% of the participants [20]. A cross-sectional, multicenter study conducted in four Swiss EDs revealed a similar trend, demonstrating that 59.6% of 69 assessed nurses accurately identified the correct triage level within the ESI triage system [21].

The PAT provides an initial impression by identifying life-threatening conditions through focused, hands-off observation to endow with immediate physiological needs and their associated level of urgency within 30-50 seconds. It is an essential component of pediatric triage, but in the current study, about 40% of the nursing officers could not acknowledge that they had ever used the PAT, and more than 70% could not expand it to its complete form correctly. While temperature and blood sugar assessments are decisive in pediatric evaluations, less than one-third of the participants comprehended their significance.

Interestingly, nursing officers in private setups demonstrated a clearer understanding of intubation during the primary survey than those in government setups. This discrepancy may arise from nursing officers’ greater independence and expertise in intubation within private establishments. In contrast, government setups often have skilled personnel in hierarchical positions who handle intubation, providing fewer opportunities for nursing officers to engage in this procedure independently. It may also be because the nursing officers in the ED in private hospitals are usually first responders, whereas resident doctors usually handle the cases in government hospitals.

A cross-sectional study in South Ethiopia, encompassing 101 nurses, revealed a need to improve triage knowledge. The study further identified significant associations between triage knowledge, experience, working experience, and educational qualification level. Consequently, the study recommended the establishment of comprehensive training programs and educational strategies to enhance triage skills and knowledge [24]. Our study showed that the group with higher degrees performed significantly better than other groups.

Contrary to the present study, a questionnaire-based investigation conducted on 266 emergency room nurses across four secondary and tertiary healthcare centers in Indonesia revealed a notably enhanced comprehension of triage skills. Moreover, the study unveiled statistically significant positive correlations between triage skills, working experience, training experience, and triage knowledge [25].

This study also unveils a conspicuous knowledge disparity among nursing officers concerning pediatric triaging, although a substantial majority (66%) of the participants possess at least some prior experience handling pediatric emergencies. This deficiency persists irrespective of their years of experience, educational qualifications, or employment status in government or private healthcare settings. Another cross-sectional descriptive study on 124 nursing students revealed low awareness scores in triage knowledge, but those with prior emergency exposure demonstrated better scores, highlighting the need for emphasized triage training [26]. These findings also resonate with the status of working nurses, as similar patterns emerge in their utilization of practical emergency triage.

A minor limitation of the study was the maintenance of responders’ confidentiality, which consequently abated the possibility of further analysis based on the gender of NOs/nurse participants and rural versus urban health centers. While collecting such information and maintaining it in a coded format could have been an option, it might have reduced the sample size. However, this safeguarding measure led to a substantial increase in study participation, resulting in a robust sample size exceeding 5,000 individuals. This approach empowered participants to respond candidly, free from potential biases. As it was a self-administered survey, recall bias was possible.

## Conclusions

The study revealed a notable disparity in knowledge and proficiency concerning pediatric triage among nurses in India, emphasizing its significant and indispensable correlation with essential nursing qualification, working setup, prior exposure or training on triage skills, and overall ED working experience. The study highlights the need for improvement in nurses’ knowledge about applying the PAT for initial patient evaluation, identifying primary and secondary survey components, and various types of triage systems used across the globe. Around half of the participants were never assessed for cognitive or psychomotor domains during their final or exit examination.

This study raises the need for improved and practically integrated triage training and examinations within the nursing curriculum, accompanied by targeted investment in emergency skills training focusing on emergency triage and treatment. Once adequately trained, nurses can be effectively designated as triage officers in all EDs. These measures may be crucial for strengthening ED capacity and equipping healthcare professionals to manage various health emergencies, including disasters and mass casualties, more efficiently.
